# Multi-Unit Serial Polynomial Multiplier to Accelerate NTRU-Based Cryptographic Schemes in IoT Embedded Systems

**DOI:** 10.3390/s22052057

**Published:** 2022-03-07

**Authors:** Santiago Sánchez-Solano, Eros Camacho-Ruiz, Macarena C. Martínez-Rodríguez, Piedad Brox

**Affiliations:** Instituto de Microelectrónica de Sevilla, IMSE-CNM, CSIC/University of Seville, 41092 Seville, Spain; camacho@imse-cnm.csic.es (E.C.-R.); macarena@imse-cnm.csic.es (M.C.M.-R.); brox@imse-cnm.csic.es (P.B.)

**Keywords:** IoT embedded systems, hardware security, postquantum cryptography, public-key encryption scheme, HW/SW codesign techniques, programmable systems-on-chip

## Abstract

Concern for the security of embedded systems that implement IoT devices has become a crucial issue, as these devices today support an increasing number of applications and services that store and exchange information whose integrity, privacy, and authenticity must be adequately guaranteed. Modern lattice-based cryptographic schemes have proven to be a good alternative, both to face the security threats that arise as a consequence of the development of quantum computing and to allow efficient implementations of cryptographic primitives in resource-limited embedded systems, such as those used in consumer and industrial applications of the IoT. This article describes the hardware implementation of parameterized multi-unit serial polynomial multipliers to speed up time-consuming operations in NTRU-based cryptographic schemes. The flexibility in selecting the design parameters and the interconnection protocol with a general-purpose processor allow them to be applied both to the standardized variants of NTRU and to the new proposals that are being considered in the post-quantum contest currently held by the National Institute of Standards and Technology, as well as to obtain an adequate cost/performance/security-level trade-off for a target application. The designs are provided as AXI4 bus-compliant intellectual property modules that can be easily incorporated into embedded systems developed with the Vivado design tools. The work provides an extensive set of implementation and characterization results in devices of the Xilinx Zynq-7000 and Zynq UltraScale+ families for the different sets of parameters defined in the NTRUEncrypt standard. It also includes details of their plug and play inclusion as hardware accelerators in the C implementation of this public-key encryption scheme codified in the LibNTRU library, showing that acceleration factors of up to 3.1 are achieved when compared to pure software implementations running on the processing systems included in the programmable devices.

## 1. Introduction

The rapid growth of the Internet of Things (IoT) has required a concentration of efforts in the development and deployment of efficient operational architectures to support and provide a multiplicity of new applications and services. Modern IoT devices incorporate a wide range of sensors to capture information about their surroundings, as well as a set of complex algorithms to process that information. From an implementation point of view, the design of portable IoT devices for user applications in embedded systems with limited resources imposes severe requirements in terms of computational capacity, memory, and power consumption, which poses an open research challenge for the electronic engineering community [1,2,3,4,5].

The application of IoT technologies in industrial environments (Industrial IoT, or IIoT) is also one of the main pillars of a new industrial revolution, often referred to as Industry 4.0 [6,7,8], where intelligent manufacturing systems leverage the evolution of Information and Communication Technology (ICT) to become much more efficient. IIoT enables improvements in many of the processes involved in a manufacturing plant, such as engineering, production, logistics, and management of supply chain activities, which translates into increased productivity and greater economic benefits [9,10]. IIoT devices have been developed for a wide variety of application domains including automotive, energy, electronics, aerospace, defense, and other industrial sectors [11].

For both consumer electronics and industrial systems, security has become a key issue, as attacks can exploit vulnerabilities in devices to compromise the use of critical applications, deny essential services, and even partially or permanently damage infrastructures and production lines [12,13,14,15]. In an IoT device, attackers can use the sensors to transfer malicious code or trigger a message to activate malware, capture sensitive personal data shared between devices, or reveal confidential information by capturing encryption and decryption keys [16,17]. In the context of IIoT, the impact of attacks could also have incalculable consequences on the mission, functions, image, or reputation of companies and corporations [18,19,20]. Security and privacy concerns in IoT and IIoT ecosystems must consider all layers of the service-oriented architectures commonly used to ensure efficient interoperability between a typically large number of heterogeneous and physically dispersed devices [2,21].

Algorithms based on Public-Key Infrastructure (PKI) are fundamental security primitives in cryptosystems currently used in applications and protocols that offer a guarantee of confidentiality, authenticity, and non-repudiability in the capture, storage, processing, and exchange of data on the Internet [22,23]. In the context of the Systems-on-Chips (SoCs) used in the IoT, these elements have also proven their usefulness to guarantee security in other system operation stages, such as secure boot, which prevents possible attacks carried out by injecting malware code into the flash boot memory with the objective of modifying the function of the system or supplanting its identity [24]. However, as is widely known, current PKI-based algorithms will be vulnerable to attacks from large-scale future quantum computers. Shor’s algorithm for integer factorization [25], which shows that quantum computers can be used to factor integers in polynomial time, reveals a vulnerability of the popular RSA algorithm, based on the assumed difficulty of factoring a large biprime number. Furthermore, Shor’s algorithm can also compute in polynomial time the Discrete Logarithm Problem (DLP) that is the basis for other asymmetric cryptographic schemes such as Diffie–Hellman (DH), Digital Signature Algorithm (DSA), and Elliptic Curve Cryptography (ECC) [26].

To deal with this threat, numerous efforts have been made in recent years in the search for algorithms resistant to quantum attacks (the so-called post-quantum algorithms) [27,28]. As a consequence, the National Institute of Standards and Technology (NIST) initiated in 2017 a Post-Quantum Cryptography (PQC) standardization process to develop new public-key cryptography standards to be used as quantum-resistant counterparts to existing standards, including digital signature and key establishment schemes [29]. The NIST PQC contest encompasses several rounds in which submissions are evaluated in terms of security and performance. The selection criteria for security is based on the algorithm resistance analysis against both classical and quantum attacks, whereas performance is measured on various classical platforms. Round 1 provided 69 submissions that were presented in December 2017. This initial selection was reduced to 26 candidates in Round 2 (January 2019). The NIST presented the results of Round 3 with seven potential candidates and eight alternatives for PQC in July 2020 [30]. Hardware implementations of Round 3 candidates to improve efficiency have been evaluated at the Third PQC Standardization Conference [31].

Ring Learning With Errors (Ring-LWE) is the substrate of various lattice-based post-quantum cryptosystems, such as the well-known public-key encryption scheme called NTRU (Nth Degree Truncated Polynomial Ring Unit). Proposed in 1988 by Hoffstein et al. [32], NTRU has become quite popular over the years due to the use of small key sizes and its speed compared to other cryptosystems with the same security level. The security of NTRU relies on a very hard problem in lattice reduction, known as the Shortest Vector Problem (SVP). Until now, there has been no polynomial-time algorithm to solve this problem. The NTRU public key cryptosystem was standardized in 2008 by the Institute of Electrical and Electronics Engineers in IEEE Std 1363.1-2008 [33] and in 2010 by the American National Standards Institute in ANSI Std X9.98 [34]. Throughout successive revisions, it has been progressively improved, mainly in aspects related to the selection of parameter sets to resist different types of attacks [35,36,37,38,39,40]. Four of the Round 1 submissions to the NIST PQC standardization contest were based on NTRU: NTRUEncrypt [41], NTRU-HRSS-KEM [42], and NTRU Prime [43] focused on Key Encapsulation Mechanisms (KEMs), and pqNTRUSign [44], designed to provide digital signature functionalities. The first two proposals were merged in Round 2 to give rise to a new submission (NTRU [45]), which reached Round 3 and is currently among the finalists.

As a consequence of its resistance to possible quantum attacks and its relatively low computational load (especially compared to cryptosystems based on the problems of factoring integers or finding discrete logarithms), NTRU is a good candidate to provide different functionalities related to the security of systems connected through public networks [46]. For this reason, many implementations of the NTRU encryption and decryption schemes have been proposed in the literature in the last 25 years. These proposals range from complete software implementations for embedded systems [47,48] to complex hardware solutions for high-end servers implemented on Application Specific Integrated Circuits (ASICs) [49,50] or Field Programmable Gate Arrays (FPGAs) [51]. Many of the first proposals were aimed at speeding up the most time-consuming parts of the algorithms (In particular, the operation of multiplication of polynomials in the nth degree truncated polynomial ring on which the different NTRU cryptographic schemes are based). These proposals pursued the double objective of making their implementation possible in electronic systems with few resources and to achieve higher processing speed than that offered by the low-end processors used in many IoT devices [52,53,54,55,56,57,58,59,60,61,62,63,64]. Recently, the proliferation of SoCs, largely supported by the evolution of programmable logic devices, has boosted the use of Hardware/Software (HW/SW) codesign techniques to combine flexibility and efficiency when implementing the different parts of any cryptographic algorithm [62,63]. Directly related to the progress of the NIST PQC competition, the use of this design strategy has increased in recent years as a consequence of the need to develop benchmarking procedures that allow a ‘fair’ comparison of hardware implementations of the different candidates [65,66,67]. With regard to design flows, the use of High-Level Synthesis (HLS) tools and Register Transfer Level (RTL) based approaches is currently being considered. The former certainly facilitates exploration of the design space [68], while the latter is better to take advantage of the structure and hardware resources available in a given device [69].

Taking advantage of the resources offered by modern programmable devices, such as the Xilinx Zynq-7000 SoC and Zynq UltraScale+ MPSoC, which allow combining the execution of software on a general-purpose processor with the implementation of hardware accelerators on the FPGA fabric, this paper addresses the implementation of the NTRUEncrypt cryptographic scheme on embedded systems by following an HW/SW codesign strategy. Its main contributions include the following:

- The design of a highly configurable intellectual property (IP) module to implement a multi-unit serial polynomial multiplier and accelerate NTRU operations;
- The proposal of different interconnection schemes that optimize the bandwidth of communication infrastructures provided by device manufacturers;
- The possibility of choosing the number of arithmetic units in the multiplier, as well as selecting the interconnection scheme to be used, which allows establishing an adequate cost/performance/security level trade-off based on the intended application for the embedded system.

The paper is organized as follows. Section 2 illustrates the mathematical foundations of the encryption and decryption operations defined in the NTRU public-key cryptosystem. Section 3 provides a historical review of the different proposals for implementation in embedded systems. The architecture and main functional blocks of the proposed configurable multi-unit serial polynomial multipliers, as well as their encapsulation as IP modules that can be connected to the processor system through standard buses, is described in Section 4, while Section 5 includes implementation results in terms of logical resources consumption and operating frequencies. The integration of hardware accelerators into the open source library LibNTRU is detailed in Section 6, which also provides statistics on the efficiency of the proposed approach in terms of the speed-up factor in algorithm execution. Finally, the main conclusions of the work are summarized in Section 7.

## 2. The NTRU Cryptographic Scheme

The cryptographic techniques included in NTRU use polynomial operations on a particular algebraic structure coming from the so-called polynomial quotient rings or polynomial convolution rings. A truncated polynomial ring of degree *N* is the quotient ring given by Equation (1), where $Z_{t}\left[X\right]$ is the set of polynomials with integer coefficients reduced module *t*, and $\left(X^{N}-1\right)$ is the polynomial defined in the currently standardized version to obtain the modulus of the polynomial arithmetic operations used by the different cryptographic primitives:

(1) $$R_{N,t}=\frac{Z_{t}\left[X\right]}{\left(X^{N}-1\right)}$$

A polynomial *a* in *X* is defined by a set of integer coefficients $a_{i}$, where *i* represents the coefficient of *a* of degree *i*, as shows Equation (2):

(2) $$a\left(x\right)=a_{0}+a_{1}\cdot x+a_{2}\cdot x^{2}+...+a_{i}\cdot x^{i}+...+a_{(N-1})\cdot x^{\left(N-1\right)}$$

Polynomial multiplication is required by encryption and decryption operations in NTRU-based schemes. The multiplication of two polynomials, c(x)=a(x)×b(x), is another polynomial whose coefficients are calculated according to Equation (3):

(3) $$c_{k}=\sum_{i+j=k\;mod\;N}\left(a_{i}\cdot b_{j}\right)\;mod\;t,\;\;\mathrm{for}\;i,j,k=0,\ldots ,N-1$$

NTRUEncrypt operations use two truncated polynomial rings, $R_{N,q}$ and $R_{N,p}$, where *N* is a prime number used to determine the degree of the truncated polynomials, and *q* and *p* are coprime, *q* being considerably larger than *p*(q≫p). The different parameter sets defined in the standard fix the values of *q* and *p* to 2048 and 3, respectively, so the elements of $R_{N,p}$ are ternary polynomials whose coefficients only have values equal to 1, −1, or 0. In order to provide different trade-offs between security and efficiency, the standard also fixes three integer values for each parameter set which determine the number of ones in the polynomial corresponding to the private key *f*, $\left(d_{f}\right)$; the temporary polynomial *g*, $\left(d_{g}\right)$; and the blinding polynomial *r*, $\left(d_{r}\right)$. Table 1 shows the values and recommended security level for the parameter sets defined in IEEE Std 1363.1.

As in other cryptographic schemes, the operations defined in NTRUEncrypt have basically three purposes: key generation, encryption, and decryption. During the key generation process, two ternary polynomials are randomly chosen. The polynomial $f\left(x\right)\in R_{N,p}$ must be invertible modulo *p* and modulo *q* and have $d_{f}$ coefficients equal to one and $\left(d_{f-1}\right)$ coefficients equal to minus one, while the remaining $\left(N-2d_{f}-1\right)$ coefficients will be zero. The other polynomial, $g\left(x\right)\in R_{N,p}$, is not required to be invertible. In this case, $d_{g}$ coefficients must be equal to one, $d_{g}$ equal to minus one, and $\left(N-2d_{g}\right)$ will be zero. Both f(x) and g(x) are secret polynomials that are used to derive the public key, $h\left(x\right)\in R_{N,q}$, using Equation (4):

(4) $$h\left(x\right)=p\cdot f_{q}\left(x\right)\times g\left(x\right)\;mod\;q$$

where the polynomial $f_{q}(x$) is the inverse of f(x) modulo *q*.

To perform the encryption operation, the message is encoded as a ternary polynomial, m(x), and a blinding polynomial, $g\left(x\right)\in R_{N,p}$, used to obfuscate the message is randomly chosen with $d_{r}$ coefficients equal to one, $d_{r}$ equal to minus one, and $\left(N-2d_{r}\right)$ equal be zero. In the initial NTRU proposal [32], the encrypted message, $c\left(x\right)\in R_{N,q}$ is obtained by applying polynomial multiplication and addition operations given by Equation (5):

(5) c(x)=h(x)×r(x)+m(x)modq

The version of NTRUEncrypt submitted to Round 1 of the NIST PQC contest [41] includes a padding mechanism, based on [36], to deal with potential insufficient entropy in a message. The performed operations correspond in this case to those shown in Equation (6):

(6) $$\begin{array}{c} m^{\prime }\left(x\right)=m\left(x\right)+mask\left(x\right)\;mod\;p\\ c\left(x\right)=h\left(x\right)\times r\left(x\right)+m^{\prime }\left(x\right)\;mod\;q \end{array}$$

Polynomial multiplication modulo *q* is also required by the decryption operation in NTRU-based schemes to calculate an intermediate polynomial as the product of the ciphertext and the private key, according to Equation (7):

(7) a(x)=c(x)×f(x)modq

which is in turn used to obtain the message *m* (or the padded version $m^{\prime }$ in NTRUEncrypt) using Equation (8):

(8) $$m^{\prime }\left(x\right)=a\left(x\right)\times f_{p}\left(x\right)\;mod\;p$$

The NTRU version submitted to Round 2 and currently under consideration in Round 3 of the said competition recommends the utilization of other parameter sets and requires multiplication in additional truncated polynomial rings, so the results of this work may also be applied to the new versions under evaluation.

## 3. Implementation of NTRU on Embedded Systems

As corroborated by the software execution profiles of their reference implementations, truncated polynomial ring multiplication is the most time-consuming function of NTRU and other lattice-based algorithms [67]. For this reason, efforts to accelerate these cryptographic schemes are primarily focused on providing an efficient software or hardware implementation of this operation. According to Equation (3), a polynomial multiplication in $R_{N,t}$ is given by the cyclic convolution of the coefficients of two polynomials. For example, the multiplication carried out during the encryption operation described by Equation (5) can be expressed as shown in Equation (9):

(9) $$e_{k}=\sum_{i+j=k\;mod\;N}\left(h_{j}\cdot r_{i}\right)\;mod\;q$$

**Figure 1:**
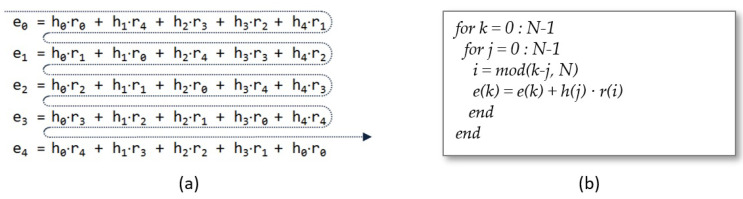
Evaluation of each term of the summation (**a**) and generation of indices *i*, *j*, and *k* (**b**) while sweeping the matrix by rows.

Figure 1a graphically illustrates the cyclic convolution of the coefficients of the polynomials h(x) and r(x) for a trivial case with N=5, while pseudocode in Figure 1b shows the algorithm for generating indices and obtaining output coefficients that would give as results a sweep of the coefficient matrix by rows. A simple and naive software implementation of this algorithm on a sequential processor would perform N·N scalar multiplications and would take the same number of clock cycles to complete the operation (assuming that only one cycle is inverted at each multiplication). This assumption is not necessarily true for low-end processors. Fortunately, the fact that the polynomial r(x) is chosen as a ternary polynomial according to IEEE Std 1363.1-2008 [33] (or even binary in the case of the early NTRU proposals [32]), allows these multiplications to be replaced by additions or subtractions of the corresponding coefficient $h_{j}$ depending on whether $r_{i}$ is 1 or −1, respectively. This was the technique used by Atici et al. in [52] for ASIC implementation in 0.13 μm technology of a compact, low-power NTRU design, with (N=167,q=128,p=3) that performs both encryption and decryption and is suitable for generalized security applications such as RFID and sensor nodes.

**Figure 2:**
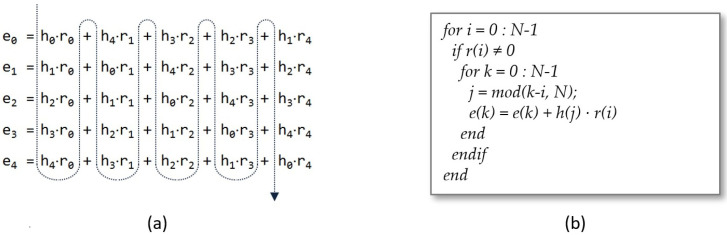
Evaluation of each term of the summation (**a**) and generation of indices *i*, *j*, and *k* (**b**) while sweeping the matrix by columns.

An additional reduction in execution time can be achieved by changing the order of generation of the indices and taking into account that r(x) is a sparse polynomial, that is, with a high number of null coefficients, which implies that there are complete columns of the convolution matrix that do not contribute to the product. Figure 2a,b show, respectively, the order of evaluation of the partial products and the pseudocode of the operation, in which it is possible to observe that the inner cycle to obtain the output coefficients is not executed when r(i) is null.

This feature was exploited by Zhan et al. in [53] to implement 2 ASICs in 0.18 μm technology that incorporate a non-zero coefficients sequence generator (NCSG) to record the indices of non-zero terms in polynomial r(x) when its coefficients are loaded into the multiplier. One of the designs is a lightweight solution that implements the lowest security level parameter set recommended in the original NTRU proposal (N=107,p=3,q=64, $d_{r}=5)$. The other one corresponds to a high-speed implementation with (N=251, p=2, $q=128,d_{r}=72)$ of the NTRU variant proposed in [35], where f=1+p*F is chosen in the key generation stage in order to eliminate a multiplication of polynomials in the decryption operation. This proposal also makes use of a property of the product of sparse polynomials analyzed in [54], which consists of defining $r=r_{1}*r_{2}+r_{3}$, with $d_{r1}+d_{r2}+d_{r3}=d_{r}$, so that the product can be carried out in three successive steps with a total duration of $(d_{r1}+d_{r2}+d_{r3})\cdot N$ clock cycles. (Note that the number of non-zero coefficients of r(x) is $d_{r}$ when p=2 and $2\cdot d_{r}$ if p=3.)

Unlike what happens in software implementations on sequential processors, polynomial multiplication can be accelerated in hardware implementations by increasing the degree of parallelism to simultaneously calculate more than one term of the convolution matrix. This was the strategy followed by O’Rourke in [55], where the author analyzed the design in TSMC 0.35 μm technology of a scalable multiplier with a variable number of parallel arithmetic units capable of implementing the highest security parameter set of the initial NTRU proposal (N=503,p=3,q=256). Another scalable solution for ultra-low power applications, which uses a circular shift register to rotate the coefficients of the polynomial r(x) during convolution multiplication, was proposed by Kaps in [56] for the parameter set (N=167,p=3,q=128). The number of clock cycles required to complete the operation is in both cases proportional to N·⌈N/n⌉, *n* being the number of parallel arithmetic units.

In systems that have sufficient resources, the time spent in the multiplication operation can be reduced to *N* clock cycles by using *N* arithmetic units in parallel. This is shown in the decryption operation proposed by Kamal and Youssef for the same parameter set in [57], which describes an NTRU implementation hardened against fault-insertion attacks on a Virtex-E FPGA. The previous value can even be reduced if the sparse nature of the polynomial r(x) is taken into account. This is illustrated by the same authors in [58], where *N* arithmetic units are used in combination with an (N,s)-Shifter, capable of shifting in each clock cycle the *N* coefficients of h(x) by a relatively small number of locations (s<<N) to obtain a multiplication time that tends to $2\cdot d_{r}$ when *s* increases in an NTRU implementation with (N=251,p=3,q=128).

The main problem with these alternatives is to provide a mechanism to supply the arithmetic units with the necessary coefficients in each cycle of operation. The parallel architecture for polynomial multiplication proposed by Liu and Wu in [59] offers an efficient and smart solution to this problem using a circuit structure similar to a Linear Feedback Shift Register (LFSR). Initially described for the implementation in a Cyclone IV FPGA of four parameter sets prior to the IEEE standard, this architecture has subsequently been taken as the basis for different improvements [60,61,62,63,64]. A timing-optimized version of this architecture is introduced by the same authors in [60], which reduces the number of clock cycles by omitting the multiplication operation when two consecutive zero coefficients are detected in the polynomial r(x). This proposal, focused only on the NTRU encryption operation, shows the resources required for the implementation in a Cyclone IV FPGA of all the parameter sets defined in the IEEE standard. Braun et al. presented in [61] a complete implementation of the standardized version of NTRU on Zynq-7000 devices, which uses the LFSR-based architecture and includes the padding scheme defined in IEEE Std 1362.1 to prevent Chosen Ciphertext Attacks (CCA). This work also reveals a certain security degradation when using the architecture proposed in [60]. The vulnerability can be minimized, while achieving a higher operating speed, with the solution proposed in [63] to design a dedicated hardware module that, at the expense of a small increase in resource consumption, is capable of detecting the presence of two, three, or four consecutive zeros in the blinding polynomial. Finally, Qin et al. in [64] presented a complete implementation of the NTRU version submitted to the third round of the NIST PQC contest using the LFSR structure to build the three types of polynomial multipliers required by the algorithm.

Despite providing the best results in terms of performance, LFSR-based solutions may not be viable for some IoT applications where programmable device resources are limited or must be used to implement other parts of the embedded system. In these cases, the availability of the multi-unit serial polynomial multiplier described in the following sections of this work provides a scalable solution that allows the selection of adequate cost/performance trade-offs.

## 4. Multi-Unit Serial Polynomial Multiplier

The resources required to implement a serial polynomial multiplier following the approaches depicted in Figure 1 and Figure 2 are practically the same, and the number of operations performed and, therefore, the execution time of the operation can be significantly reduced in the second case, since it is not necessary to execute the internal loop of the algorithm for null values of r(i). In this way, the number of clock cycles required to complete the operation is reduced from N·N to $N\cdot 2\cdot d_{r}+\left(N-2\cdot d_{r}\right)$, where the term in parentheses corresponds to the number of times a zero value is obtained when accessing the memory that stores the coefficients r(i). For the parameter sets proposed in the IEEE standard, this translates into a reduction in operating time that varies between 43.5% and 89.4% (for EES401EP1 and EES1499EP1, respectively).

**Figure 3:**
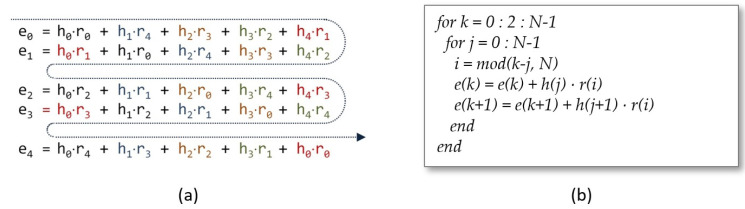
Simultaneous evaluation of two terms of the summation (**a**) and generation of indices *i*, *j*, and *k* (**b**) when the convolution matrix is swept by rows (colors indicate the terms evaluated in parallel).

In addition, for both alternatives, the response time of the multiplier can be improved up to a factor *M* by replicating *M* times the arithmetic unit to calculate in parallel *M* partial terms of the convolution matrix. For this, however, it is necessary to ensure that the appropriate *M* coefficients h(j) are available in each cycle of the algorithm and to guarantee that the memory locations containing the partial results e(k) involved in the operation can be accessed. When the convolution matrix is swept by rows, the memories that contain the coefficients of the input polynomials do not need to be modified if the terms that are calculated simultaneously share the value of r(i). As can be observed in Figure 3 for M=2, in this case, it is only necessary to consider a new coefficient h(j) in each clock cycle, so a shift register of length *M* can be used to provide the *M* coefficients, h(j)⋯h(j+M−1), necessary to calculate the *M* coefficients e(k)⋯e(k+M−1).

**Figure 4:**
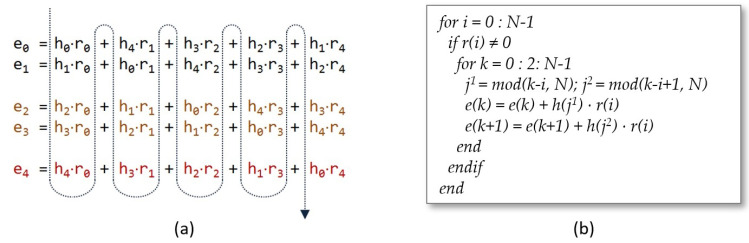
Simultaneous evaluation of two terms of the summation (**a**) and generation of indices *i*, *j*, and *k* (**b**) when the convolution matrix is swept by columns (colors indicate the terms evaluated in parallel).

On the contrary, if *i* is chosen as the index of the outer loop, as illustrated in Figure 4, *M* coefficients h(j) must be provided simultaneously. These coefficients follow a correlative order, but the initial element in each column varies as a function of the values of *i* and *k*. As *i* increases by one unit in each run of the outer loop and *N* is always a prime number, it is not possible to find a value of *M* that allows for grouping the coefficients in a single memory capable of providing the possible combinations of *N* consecutive elements in each clock cycle taken from *M* in *M*. The solution adopted in this proposal consists of replicating *M* times the memory that stores the coefficients h(j) and providing a mechanism that facilitates the loading of the coefficients in the appropriate order during the initial phase of the algorithm execution.

### 4.1. Core Design of the Polynomial Multiplier

**Figure 5:**
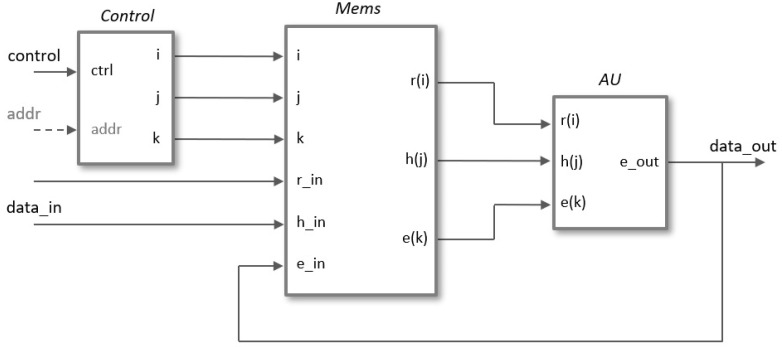
Block diagram for the serial implementation of the polynomial multiplier required in NTRU.

A generic architecture for the implementation of a scalable polynomial multiplier, capable of supporting different degrees of multiplicity and adaptable to the parameter sets proposed in the literature, is described in this section. The module has been developed using an HDL design flow provided by Xilinx Vivado tools, in which all components are described in Verilog using parameter and generate blocks to guarantee its configurability. The simplified block diagram in Figure 5 shows the main functional blocks necessary for the hardware implementation of the polynomial multiplication operation.

The *Control* block generates the indices *i*, *j*, and *k* in the different operation phases (*Load* coefficients, *Operate*, and *Read* result). These indices act as the addresses of the memories included in *Mems*, which contain the coefficients of the input polynomials and the partial and final results of the output polynomial. The arithmetic unit, *AU*, adds/subtracts the value of h(j) to/from the current partial value e(k) depending on whether the coefficient r(i) is 1 or −1. The width of the buses depends on both the security level chosen for the NTRU algorithm (parameter set) and the degree of multiplicity used (value of *M*).

**Figure 6:**
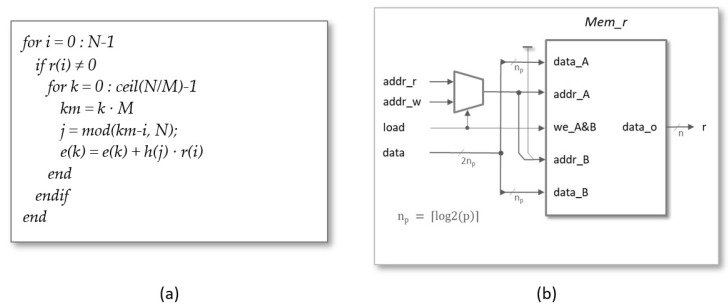
(**a**) Pseudocode used to generate the indices for multiple arithmetic units. (**b**) Memory block used to store the coefficients of the polynomial r(x).

The pseudocode shown in Figure 6a can be used to generate the indices in the generic case of using *M* arithmetic units to perform the polynomial multiplier operation. The *i*-index is used in the outer loop to sweep the multiplication matrix by columns to be able to eliminate those in which r(i)=0, and the *k*-index is used in the inner loop in such a way that the successive values of *i* also determine the memory addresses of the *M* terms to be calculated in parallel. The *j*-index corresponding to the first of the coefficients h(j) to be evaluated in parallel is calculated employing a modulo *N* operation between the values of km=k·M and *i*. As the execution of the internal cycle of the algorithm depends on the value of r(i), it is necessary to access the aforesaid value in the same clock cycle in which it is decided whether or not to increase the counter *i*. To fulfill this condition, in the HDL description, the counters *i* and *k* increase with the decrease in the clock signal while the rest of the system operates with the rising edge of the clock signal.

The *Mems* block groups the memory structures used to store the coefficients of the input polynomials, r(i) and h(j), as well as the partial and final values of the coefficients e(k) of the polynomial resulting from the multiplication operation. The three blocks are implemented as dual-port memories using block RAM (BRAMs) available in programmable devices from different manufacturers.

Regardless of the degree of multiplicity, a single memory with N cells of $n_{p}=\left\lceil log2\left(p\right)\right\rceil$ bits is always used to store the coefficients of the blinding polynomial. As illustrated in Figure 6b, during the load phase, both port-A and port-B are used to store the values of the two coefficients simultaneously supplied by the input data bus in the memory addresses *i* and i+1, respectively. In this way, it is possible to double the bandwidth of the communication channel used to connect the hardware accelerator to the processor. On the other hand, port-A is used during the operation phase to supply the successive coefficients r(i) to the arithmetic units. A multiplexer controlled by the load signal is in charge of selecting the memory addresses in each phase of operation.

**Figure 7:**
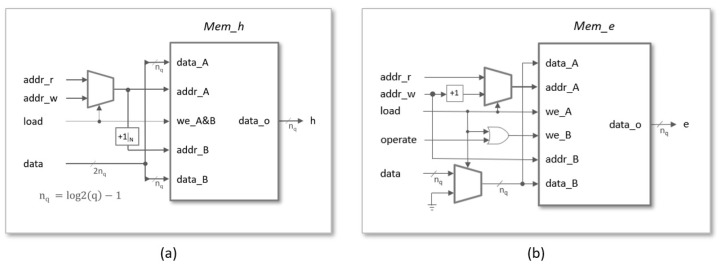
Basic memory blocks used to store the coefficients of polynomials h(x) (**a**) and e(x) (**b**).

**Figure 8:**
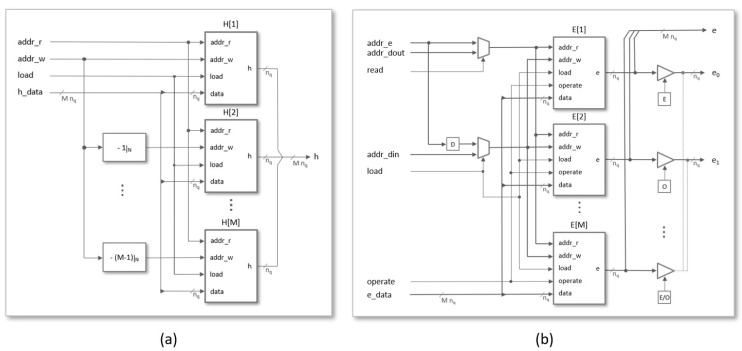
Memory structures used to store the coefficients of polynomials h(x) (**a**) and e(x) (**b**).

As shown in Figure 7a, the basic memory block used to store coefficients h(j) is similar to the previous one, but now the word length is $n_{q}=\left\lceil log2\left(q\right)\right\rceil$ and the address input of port-B is obtained by means of a block that increments the input of port-A by one and calculates the result modulo *N*, since even and odd terms are interchanged in successive replicas of the memory. In systems with multiple arithmetic units, the basic memory block must be replicated *M* times, as illustrated in Figure 8a. The copies share all the inputs, except the one corresponding to the write address bus, which is calculated as [addr(j)=addr((j−m)modN)form=0⋯M−1]. This mechanism allows the same data h(j) to be stored simultaneously at the appropriate position of the different memory replicas. As can be observed, the data are stored in pairs during the load phase and read from *M* to *M* during the operation phase.

The basic memory block for the coefficients e(k), shown in Figure 7b, has some differences compared to the previous two. The main one is that its size is now only ⌈N/M⌉$n_{q}$-bit cells since, as mentioned before, although the total storage space remains unchanged, the proposal uses *M* memories of these dimensions to enable the calculation of *M* terms of the convolution matrix in parallel. As in the previous cases, this design also includes two multiplexers and some logic blocks to control access to the memory in each phase of operation. When the load signal is activated to store the coefficients of the multiplier input polynomials, the memory cells with addresses addr_w and addr_w+1 are initialized to zero through the data input of port-B and port-A. For this, the addr_w bus is connected to the ⌈log2(⌈N/M⌉)⌉ most significant bits of the address input supplied from the multiplier access interface. Later, during the multiplier operation phase, the value stored at the addr_r address of each memory is read by port-A and sent to an arithmetic unit, whose output (the accumulated result for the corresponding coefficient) will be stored at the same address in the next clock cycle using port-B.

The complete memory structure to store the temporal and final coefficients of the polynomial e(k) is illustrated in Figure 8b. A multiplexer controlled by the load signal is used to connect addr_w to the input address provided by the interface (addr_din) during the load phase. In the operation phase, the data stored in the cell addressed by addr_e are read to be processed by the corresponding arithmetic unit and stored, in the next clock cycle, at the same memory address. Finally, during the read phase, addr_w is connected to the output address provided by the interface (addr_dout) through a multiplexer controlled by the read signal. The block has three outputs. Output *e* is a bus of $M\cdot n_{q}$ bits, made up from concatenation of the outputs of the individual memories, used to provide *M* coefficients of e(k) to the arithmetic units in each clock cycle. Outputs e0 and e1 are $n_{q}$-bit buses used in the read phase to provide even and odds memory positions to the output interface.

**Figure 9:**
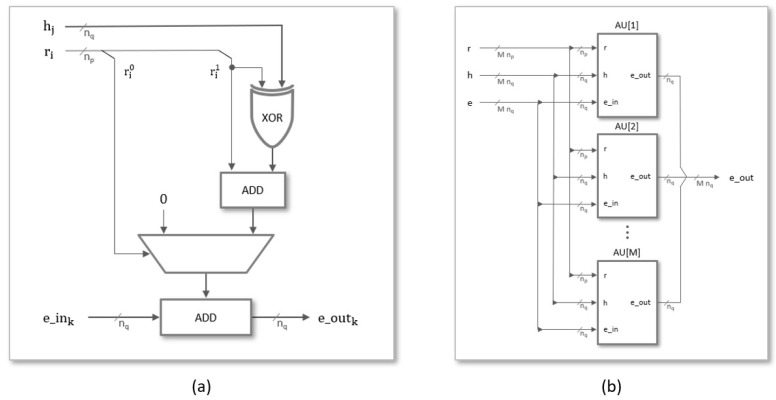
Block diagram of the arithmetic unit (**a**) and grouping of *M* arithmetic units to calculate *M* terms of the multiplier operation in parallel (**b**).

The third block of Figure 5 contains the arithmetic units in charge of evaluating the terms that appear in the summation of Equation (3). As shown in Figure 9a, the fact that one of the polynomials involved in the operation is ternary allows simplifying the implementation of this element by reducing its functionality to add or subtract from the previously accumulated value of e(k) the value of the coefficient h(j) if the value of r(i) is 1 or −1, respectively. For a polynomial multiplier of degree of multiplicity *M*, *M* of these elementary blocks must be combined according to the scheme illustrated in Figure 9b, where the input and output buses are composed by concatenating the corresponding buses of each of the arithmetic units.

### 4.2. Interface Design and IP-Module Encapsulation

In addition to the way in which the coefficients of the input polynomials are stored so that they can be properly accessed in each clock cycle, another aspect of great importance that distinguishes the different proposals for hardware-implemented polynomial multipliers is the procedure used to interact with the rest of the cryptosystem. In the case of hybrid HW/SW implementations for embedded systems, such as the one described in this work, the different functions of the cryptosystem will be executed in software on the general-purpose processor available in the system. The connection between the processor and the hardware accelerator will be made using standard interconnection buses to facilitate design reusability. In order to use the proposed multiplier as a peripheral of the ARM processors integrated into the Xilinx Zynq-7000 and Zynq UltraScale+ devices, two options based on the Advanced Extensible Interface (AXI) bus that provide different cost/performance ratio are described and compared below.

#### 4.2.1. AXI4-Lite Option

**Figure 10:**
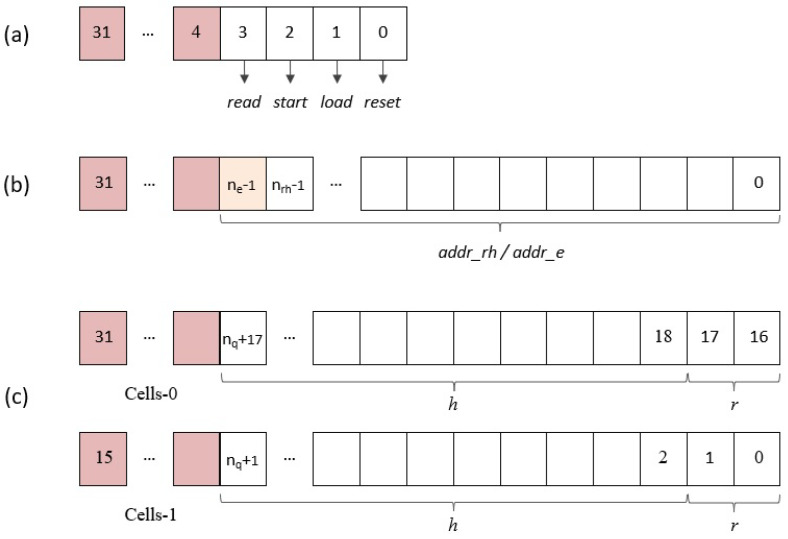
AXI4-Lite IP module input registers: (**a**) control register; (**b**) address register; and (**c**) data input register.

**Figure 11:**
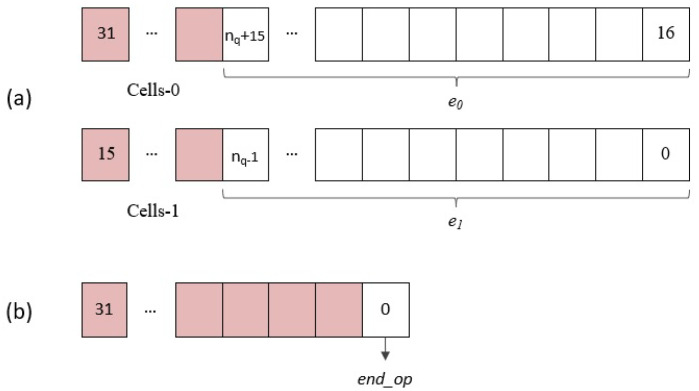
AXI4-Lite IP module output registers: (**a**) data output register and (**b**) end operation register.

This option considers the use of AXI4-Lite buses to access the registers that store the coefficients of the input and output polynomials, as well as a control register to sequence the different execution phases of the accelerator module, and a status register to detect the end of the multiplier operation:

- Control register: The four least significant bits of this input register, as shown in Figure 10a, are used from the software to supply the module reset signal (reset) and the enable signals for initialization and coefficient loading (load), operation start (start), and reading of results (read).
- Address register: During the load phase, the register shown in Figure 10b is used to indicate the indices of the coefficients r(i) and h(j), whose values are provided through the data_in register. The number of bits required to encode the memory addresses depends on the implemented parameter set $\left(n_{rh}=\left\lceil log2\left(N\right)\right\rceil \right)$. On the other hand, in the read phase, the content of this register points to the memory address of the coefficient r(k), whose value is output through the data_out register. As a consequence of the simplified addressing scheme used in the design, the number of bits needed to encode the memory addresses is, in this case, a function not only of *N* but also of the degree of multiplicity of the polynomial multiplier $\left(n_{e}=\left\lceil log2\left(N/M\right)\right\rceil +\left\lceil log2\left(M\right)\right\rceil \right)$. (When *M* is different from 1, it is necessary to generate the enable signals of the *M* memories used to calculate the result of the operation. The solution adopted in this case to simplify the generation of these signals is to generate the memory addresses externally).
- Data input (data_in) register: Considering that all the parameter sets defined by the IEEE standard use values of *p* and *q* equal to 3 and 2048, respectively, 2 pairs of coefficients r(i) and h(j) can be transmitted simultaneously in each AXI4 transfer using the bit distribution shown in Figure 10c, where ⌈log2(p)⌉+⌈log2(q)⌉ bits in the upper and lower half of the 32-bit register are used for each pair of coefficients.

- Data output (data_out) register: As in the case of input polynomials, two coefficients of the multiplier result can be retrieved in each read access to this register through the AXI4 interface. As shown in Figure 11a, $n_{q}$ bits are used in the upper and lower half of the 32-bit register.
- End operation (end_op) register: The least significant bit (LSB) of the register shown in Figure 11b gives access to the status signal of the same name, which will be used by the general-purpose processor to determine when the polynomial multiplier has finished its operation and start the results reading phase.

#### 4.2.2. AXI4-Stream Option

**Figure 12:**
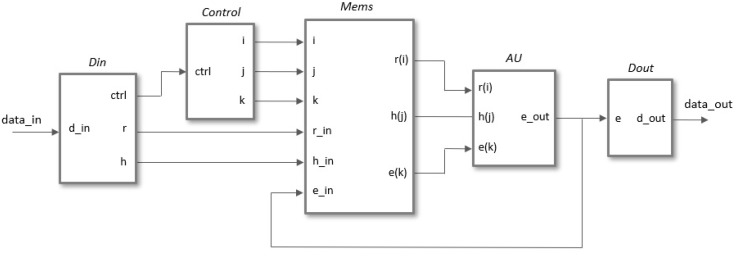
Inclusion of input and output interfaces for connecting the multiplier IP module through AXI4-Stream buses.

When using AXI4-Lite, it is necessary to send, through the address register provided by the interface, the memory addresses where the coefficients of the input polynomials and those of the multiplication result should be stored or read from. This fact, together with the need to carry out many individual transfers, can mean that even though the bandwidth of these transfers is optimized, the time required to send the multiplier operands and receive the result can limit the operation of the hardware accelerator for certain applications. Since the coefficients of the different polynomials are normally stored in or retrieved from consecutive memory locations, the most appropriate solution is to use AXI4-Stream interfaces, which can be connected to the processor via First-In First-Out (FIFO) structures or Direct Memory Access (DMA) modules to establish dedicated data paths between the HW and SW parts of the embedded system. As shown in Figure 12, to provide the multiplier IP module with AXI4-Stream interfaces, it is necessary to include two new blocks responsible for generating protocol signals of external buses, as well as internally providing memory write addresses for coefficients r(i) and h(j) in the load phase, and read addresses of coefficients e(k) during the read phase.

## 5. Implementation Results

Experimental validation and characterization of our proposals for the Xilinx Zynq-7000 and Zynq UltraScale+ device families have been carried out using the development boards Pynq-Z2 and Ultra-96, respectively. In addition to a number of generic and specific logic resources, the former includes as processing element a dual-core ARM Cortex-A9 application processor, while the latter provides a quad-core ARM Cortex-A53 application processor together with a dual-core ARM Cortex-R5 real-time processor. The tools provided by the Xilinx Vivado Design Suit were used for the implementation of the polynomial multiplier IPs from the Verilog descriptions of their functional blocks, as well as for the hardware development of embedded systems that include these IPs.

**Figure 13:**
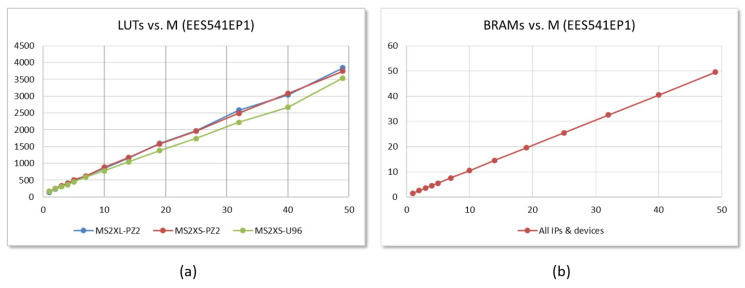
LUTs (**a**) and Block RAMs (**b**) required to implement the MS2XL and MS2XS hardware accelerators on Zynq-7000 and Zynq UltraScale+ devices for the parameter set EES541EP1 and different values of *M*.

Figure 13 illustrates the resources consumed by the implementations of the two polynomial multiplier options for the EES541EP1 parameter set and different values of *M* on Zynq-7000 and Zynq UltraScale+ devices. In order to analyze the contribution to resource consumption of each of the multiplier functional blocks, the tool option for maintaining the design hierarchy was used during the synthesis process. In the graphs, it can be seen that the number of used Look-up Tables (LUTs) increases linearly with the value of *M* for the two IPs. For a value of M=10, the consumption is less than 2% of the resources of this type available in the Zynq-7000 device and 1.8% of those available in the Zynq UltraScale+. As shown in Figure 13a, the resource consumption for both IPs is very similar. The option that uses AXI4-Stream buses requires 15 LUTs to implement the Din block and between 20 and 30 for Dout. However, this increase is practically offset by a reduction in the resources of the memory block for coefficients h(j). The data obtained for the considered parameter set show that Zynq UltraScale+ designs consume an average of 5% fewer LUTs than those implemented on Zynq-7000 devices. As shown in Figure 13b, as a consequence of the need to replicate the memory that stores the coefficients h(j), memory consumption also increases linearly with the value of *M*. As an example, the consumption of BRAMs for M=10 represents 7.5% and 4.9% of the resources of this type available in the Pynq-Z2 and Ultra-96 boards, respectively.

**Figure 14:**
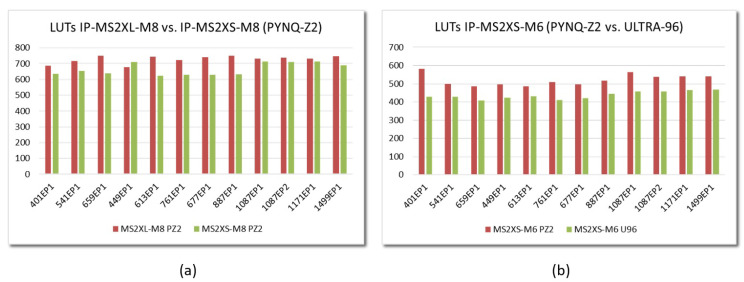
Comparison of LUTs required to implement the different IEEE Std 1363.1 parameter sets for (**a**) MS2XL and MS2XS IPs with M=8 on Zynq-7000 devices; and (**b**) MS2XS IPs with M=6 on Zynq-7000 and Zynq UltraScale+ devices.

The results are similar for the rest of the IEEE Std 1363.1 parameter sets. Figure 14a compares the data corresponding to the implementation of the two types of accelerator with eight arithmetic units (M=8) in Zynq-7000 devices. In this case, by allowing the Vivado tool to flatten the design hierarchy to optimize synthesis, the implementation of MS2XS-M8 multipliers requires, on average, almost 10% fewer resources than for MS2XL-M8 modules. On the other hand, Figure 14b allows us to compare the implementation results for MS2XS-M6 accelerators, with six arithmetic units (M=6), in the two families of programmable devices analyzed in this work. As can be seen, the use of Zynq UltraScale+ devices requires an average of 16% fewer resources than the Zynq-7000.

The encapsulation of the two designs as IP modules was carried out with the help of Xilinx tools. In both cases, Verilog descriptions include parameters to define the values of the parameters (*N*, $d_{r}$, *p*, and *q*) defined by NTRUEncrypt, as well as the degree of multiplicity, *M*, used for the synthesis and implementation of the multipliers. The IP modules can then be incorporated into the Vivado IP catalog and used like the rest of Xilinx elements to build embedded systems using the IP Integrator tool. To facilitate the design task, the configuration interfaces of both IP modules have been defined in such a way that it is possible to globally select the values for each parameter set defined in the IEEE standard or to do it independently to test new configurations.

## 6. Embedded System Integration

The interconnection of the hardware accelerator IP modules that use the AXI4-Lite interface with the processor systems of the programmable devices included in the development boards only requires the communications infrastructure provided by the Xilinx *AXI Interconnect* block. On the other hand, when the IP modules are equipped with AXI4-Stream interfaces, these must be connected to the processor systems via *AXI4-Stream FIFO* or *AXI4-Stream DMA* blocks. This last option, which also requires the use of an *AXI Smart Connect* block, has been used in the design described in this work.

### 6.1. Resource Consumption

**Figure 15:**
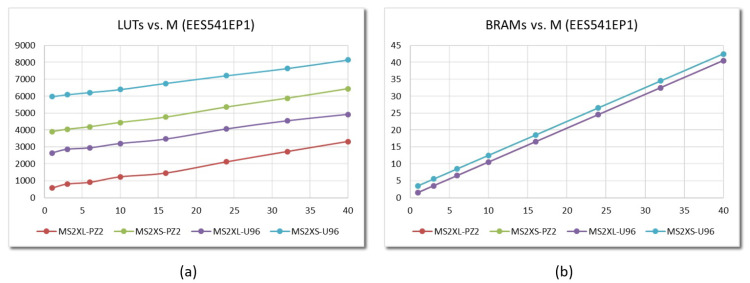
LUTs (**a**) and BRAMs (**b**) required to implement embedded systems that incorporate MS2XL and MS2XS multipliers on Zynq-7000 and Zynq UltraScale+ devices for the parameter set EES541EP1 and different values of *M*.

The cost in terms of resources required to implement polynomial multipliers with the two interface types in the two programmable devices is quite different, as is the performance in terms of speed of operation, provided by each of the alternatives. This characteristic is evidenced by the data summarized below, which correspond to the implementation in both development boards of embedded test systems that incorporate the hardware accelerator IP modules analyzed in the previous section. Figure 15a shows the dependence of the number of LUTs with the value of *M* for embedded systems incorporating MS2XL and MS2XS IPs that implement the EES541EP1 parameter set on the programmable devices of Pynq-Z2 and Ultra-96 development boards.

Comparing the graphs shown in Figure 15a with those that appeared in Figure 13a, it can be seen that the linear dependence of the number of LUTs with the value of M is maintained. Likewise, it is observed that the amount of resources needed to connect the IP to the processing system is practically independent of the degree of multiplicity of the multiplier but varies greatly depending on the type of interface and the device used. Specifically, the average values for the number of LUTs used to interface the multiplier with the processing system are 282 (MS2XL) and 3586 (MS2XS) for implementations on the Pynq-Z2 development board, increasing to 2395 (MS2XL) and 5656 (MS2XS) when using the Ultra-96 board. On the other hand, as shown in Figure 15b, the behavior regarding the number of Block RAMs is identical for both boards (graphs are overlapped) but varies depending on the type of interface used by the multiplier. When using the MS2SX option, it is necessary to add the memory units required to implement the input and output FIFOs of the AXI-DMA block used to access the IP AXI4-Stream interfaces (2 additional Block RAMS for this set of parameters).

**Figure 16:**
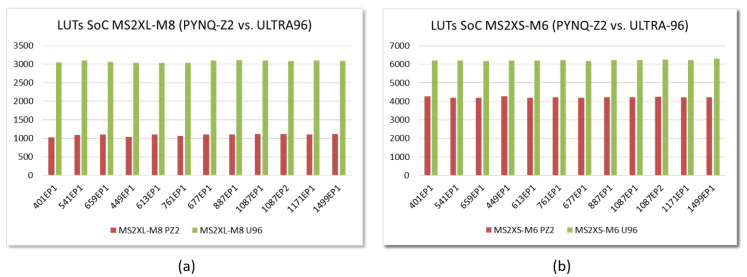
LUTs required to implement embedded systems that incorporate MS2XL-M8 (**a**) and MS2XS (**b**) multipliers on Zynq-7000 and Zynq UltraScale+ devices for the different IEEE Std 1363.1 parameter sets.

An analogous behavior can be observed for the rest of the parameter sets defined in IEEE Std 1363.1. The graphs in Figure 16 compare the implementation in the two development boards of embedded systems that incorporate multiplier IPs with the same type of interface and a given value of *M*. As can be observed, the consumption of LUTs depends both on the type of interface and the device, but it is very similar for all parameter sets. Regarding the consumption of Block RAMs, the behavior shown in Figure 15b is maintained, although it should be noted that, depending on the size of the memories provided by the programmable devices, the number of blocks increases when the value of *N* is greater than 1024 (as in the last 4 parameter sets in Table 1).

Finally, in order to illustrate in more detail the resources used and facilitate the comparison of the different interfaces and implementation platforms, Table 2 shows the resources consumed by the test systems using the two proposed multipliers with a multiplicity degree of eight to implement the EES541EP1 parameter set on two devices from the Xilinx Zynq-7000 and ZynqUltraScale+ families. In all cases, both the data related to the multiplier (IP) and the complete test system (SoC) are shown. The total amount of available resources of each type is also included (in parentheses) in the table headers so that the degree of occupation of the device can be easily estimated.

### 6.2. Performance Evaluation

In hybrid HW/SW solutions implemented in embedded systems, software components play a key role in verifying the functionality and evaluating the performance of hardware accelerators. The two development boards used in this work support the Python Productivity for Zynq (PYNQ) environment [70]. This environment provides a Python framework (running on an embedded Linux operating system) that simplifies the integration of hardware modules and their interaction with software components. To avoid the possible negative impact on operation speed caused by the use of an interpreted programming language, such as Python, in this occasion, we have used as an alternative the C-API provided in [71], which provides a similar functionality by means of a set of C routines that can be compiled to generate executable code.

Components of the PYNQ C-API provide facilities for loading bitstreams to define the functionality of programmable devices, as well as to interact with external devices through General-Purpose Input/Output (GPIO) interfaces, and with hardware blocks implemented on the programmable logic of Zynq devices by using memory-mapped and shared memory mechanisms. It also includes functions to facilitate management of the Processing System (mainly, access to internal ARM CPU registers and clock generators) and to interact with commonly used Xilinx IP blocks (such as *AXI DMA* or *AXI Interrupt Controller*). The use of these facilities simplifies not only the coding of the software drivers necessary to control the operation of the hardware multipliers but also the programming of the series of tests used to check and characterize their operation.

The driver for AXI4-Lite IPs uses read/write instructions to access the memory-mapped registers described in Section 4.2 in order to sequence the successive multiplier operation phases, as well as to load the input coefficients and read the resulting ones when the operation is completed. This driver also performs the generation of correct I/O memory addresses according to the value of *M*. Operation sequencing and coefficient addresses generation are performed internally in AXI4-Stream IPs. In this case, the driver is responsible for initializing the shared memory, configuring the DMA block, activating the operation of the read and write channels, and waiting for the operation to complete.

The IP test programs developed in this work combine the previous drivers with a set of higher-level functions that facilitate checking the multipliers behavior and obtaining metrics to compare their performance. The commands used to run them admit a series of options to choose the IP multiplicity degree (which determines the bitstream that will be used to program the Zynq device), set the number of times the test will be executed (to facilitate obtaining statistical values), and select the debug level (which conditions the output provided by the program). Using these commands through a set of shell scripts executed in the processing systems of the programmable devices included in the Pynq-Z2 and Ultra-96 boards, an extensive set of tests of the proposed solutions has been carried out, whose main results are summarized in the following paragraphs.

**Figure 17:**
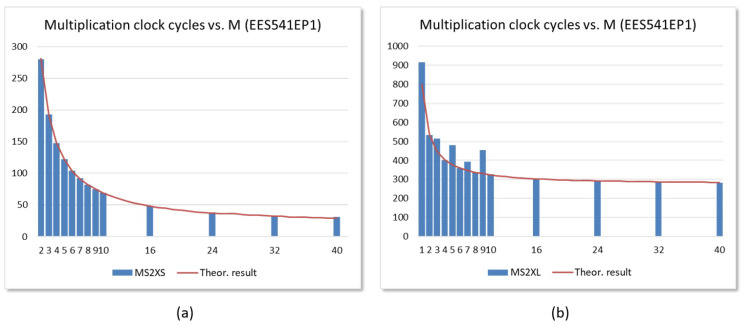
Clock cycles required to complete polynomial multiplication for embedded systems that incorporate IPs MS2XS (**a**) and MS2XL (**b**), implementing the EES541EP1 parameter set with different values of *M* in a Zynq-7000 device.

The graphs in Figure 17 show the number of clock cycles spent to complete the polynomial multiplication required in NTRUEncrypt when using embedded systems, with the two described IPs and the parameters set EES541EP1, to implement polynomial multipliers with different numbers of arithmetic units in a Zynq-7000 device.

As can be seen, the behavior varies significantly depending on the interface used by the IP module, as a consequence of the mechanisms used to load the input polynomial coefficients and read the output polynomial coefficients in both alternatives. When the MS2XS module is used, the timing response of the test systems implemented on either of the two development boards follows the theoretical curve shown with a continuous line in Figure 17a, to which a constant value corresponding to the exchange of coefficients through the AXI4-Stream interfaces has been added (approximately 10 clock cycles for the chosen parameter set).

The timing behavior of test systems that incorporate the MS2XL module presents two distinctive characteristics, as illustrated in Figure 17b for the design implemented in the Pynq-Z2 board. On the one hand, only the results of the systems that use multipliers with even values of *M* fit the theoretical curve. On the other hand, the offset that needs to be added to adjust these results is much higher in this case. The first peculiarity has to do with the selection mechanism of the different replicas of $mem_{h}$, as well as the fact that the values of two coefficients are transmitted at the same time, which means that the maximum bandwidth cannot be used when *M* takes odd values. The second is a consequence of the high number of accesses that must be made to the internal registers of the IP module through the AXI4-Lite interface to provide the operands and obtain the result of the operation. When working at the same clock frequency, implementations on the Ultra-96 board are slower than those on the Pynq-Z2 board, which is a consequence of UltraScale+ devices having a high read latency through the AXI bus and which makes the times for reading the results of the multipliers eight times greater than those for loading the coefficients.

**Figure 18:**
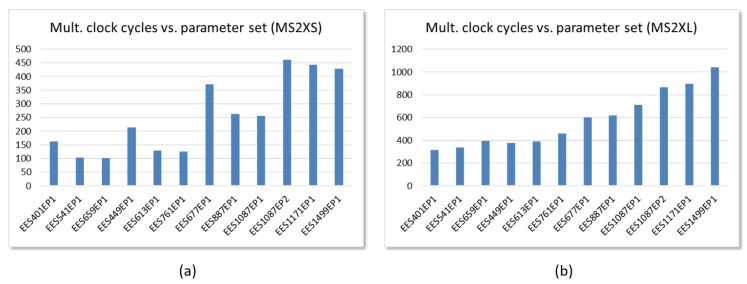
Clock cycles required to complete polynomial multiplication using the different IEEE Std 1363.1 parameter sets for embedded systems that incorporate: (**a**) MS2XS IPs with M=6 implemented in a Zynq UltraScale+; (**b**) MS2XL IPs with M=8 implemented in a Zynq-7000 device.

The behavior described is similar for other parameter sets. Operation times depend on the type of IP interface and the values of *N* and $d_{r}$ defined by the set of parameters implemented, as can be clearly seen in Figure 18a, which shows the data corresponding to the implementation on the Ultra-96 board of test systems that include the MS2XS IP with M=6 for the different sets of parameters in Table 1. However, as can be seen in Figure 18b, which compares Pynq-Z2 implementations of the IEEE std 1363 parameter sets, loading coefficients and reading result times play an important role in MS2XL IP-based test systems, which can severely affect their performance.

The high read latency through the AXI bus that affects Zynq UltraScale+ devices means, for example, that implementations based on the MS2XL IP on the Ultra-96 board have lower performance than on the Pynq-Z2 board when using clocks with the same frequency.

**Figure 19:**
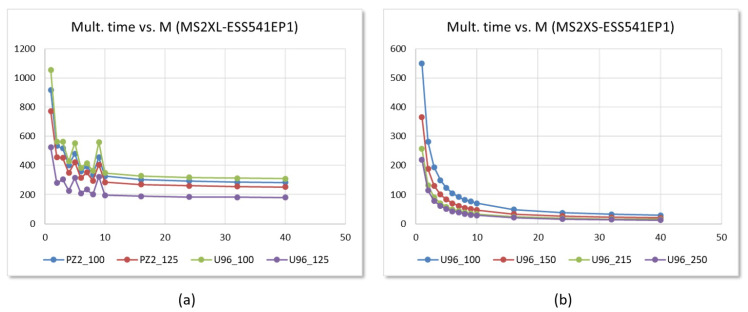
Comparison of multiplier operating times vs. *M* for: (**a**) MS2XL-based test systems implementing the parameter set EES541EP1 running at two clock frequencies on Pynq-Z2 and Ultra-96 boards and (**b**) MS2XS-based test systems implementing the same parameter set on the Ultra-96 board running at different clock frequencies.

The PYNQ environment allows to modify on the fly the frequency of clocks generated by the ARM processors in Zynq devices. This feature makes it possible to verify the behavior of the test systems under operating conditions more demanding than those initially proposed, without the need to go through the design synthesis and implementation stages again. Figure 19 illustrates some of the results of this analysis. Figure 19a shows a comparison of the relationship between the operation time and the number of arithmetic units of systems using the MS2XL IP and the EES541EP1 parameter set for different development boards and operating frequencies. The results show that when operating at 100 MHz, the implementations in Pynq-Z2 are faster than those in Ultra-96 as a consequence of the result reading times through the AXI4-Lite bus. However, the maximum clock frequency for correct operation in the Pynq-Z2 board is 125 MHz, while, with the Ultra-96, this value goes up to 250 MHz, resulting in lower times.

Figure 19b, on the other hand, also shows the relationship between operating time and *M* in systems with the same parameter set, now using the IP MS2XS, implemented on the Ultra-96 board, and operating with different clock frequencies. The results obtained show that all the test systems operate correctly when working at frequencies of 100, 150, and 215 MHz. The multiplication results are also correct for systems with M<10 using a clock of 250 MHz.

## 7. Integration of HW Accelerators into LibNTRU

The previous analysis allows us to characterize and compare, in terms of cost and performance, the implementation of the proposed polynomial multiplier on two different programmable device families as a function of the kind of interface, the degree of multiplicity, and the parameter set chosen. This information is valuable from the point of view of the implementation of the embedded system. However, from the perspective of its practical application, it is also very interesting to evaluate the level of improvement that these hardware accelerators provide when they are used to implement the cryptographic scheme for which they were conceived. To this end, this section describes the use of the proposed multipliers integrated within the LibNTRU software library [72], as well as the procedures used to estimate the improvement they provide against a fully software implementation when performing the encryption operation defined in NTRUEncrypt.

LibNTRU is a C implementation of the public-key encryption scheme NTRUEncrypt listed in IEEE Std P1363.1. It contains open source code that has been tested on different operating systems, including Windows (MinGW), Mac OS X, and Linux, with packages available for the most common distributions of the latter. In addition to the functions to implement the operations of the cryptographic scheme, LibNTRU also provides a series of test routines that simplify the verification of correct behavior and the evaluation of performance for different options and parameter sets. Version 0.5 of the library, downloaded from the GitHub repository, was used in this work in combination with the Vivado software development tools to compile and install this software into the PYNQ environment with the objective of using it for the implementation of NTRUEncrypt in embedded systems built on Xilinx’s Zynq-7000 and Zynq UltraScale+ programmable devices.

To incorporate the functionality provided by LibNTRU into the test systems described in the previous section, as well as to analyze the advantages that the use of the hardware accelerators proposed in this work can bring, it was necessary to carry out the following tasks. First, the software drivers discussed in the previous section were adapted so that they presented the same formal parameters as the library functions responsible for polynomial multiplication. Next, taking as a model those already included in the library, new test programs were coded to compare pure software implementations with hybrid solutions that perform polynomial multiplication on hardware. Finally, to facilitate migration to different development platforms and embedded systems, the makefile used to compile and install the functions and test programs was conveniently updated to incorporate the new functionality. As in the case of the test programs described in Section 6, those incorporated into the LibNTRU library also admit a set of parameters that allow the choice of the number of tests to be carried out, the times each test is repeated to obtain average values, and the amount of information provided for each of the embedded systems considered in our study. This information allows us not only to verify the correct operation of the system but also to determine the time invested in each of the tasks it performs.

In order to evaluate the performance of the hardware accelerators as a function of the number of arithmetic units, as well as to compare the behavior when using different parameter sets, more than 120 test systems were implemented on the 2 development boards.

**Figure 20:**
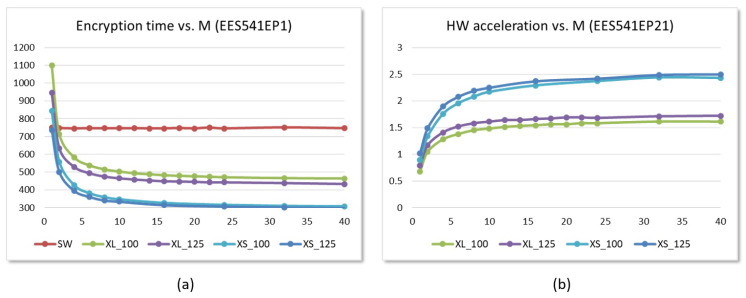
Evolution versus number of AUs of the time invested (**a**) and the acceleration factor reached (**b**) in the encryption operation using the set of parameters EES541EP1 for SW and HW/SW embedded systems implemented on the Pynq-Z2 board (time displayed in μs).

Figure 20a shows the evolution versus *M* of the time invested in NTRU encryption operation in embedded systems that use the LibNTRU library and the two IPs proposed in this work. The graphs allow us to compare the results of the software implementations executed on the ARM Cortex-A9 processors of the Zynq-7000 device on the Pynq-Z2 board with hybrid HW/SW implementations using AXI4-Lite and AXI4-Stream versions of the polynomial multiplier controlled by clock signals of 100 and 125 MHz. As can be seen in Figure 20b, for the set of parameters chosen, the use of both hardware accelerators allows improving the performance of the encryption operation for values of M≥2. *The acceleration factors* range *from 1.05* (for the AXI4-Lite-based IP with M=2 @ 100 MHz) *to 2.5* (for the AXI4-Stream IP with M=40 @ 125 MHz). For a typical value of M=10, the acceleration factors range *from 1.48 to 2.25*, depending on the interface of the multiplier used and its operating frequency.

**Figure 21:**
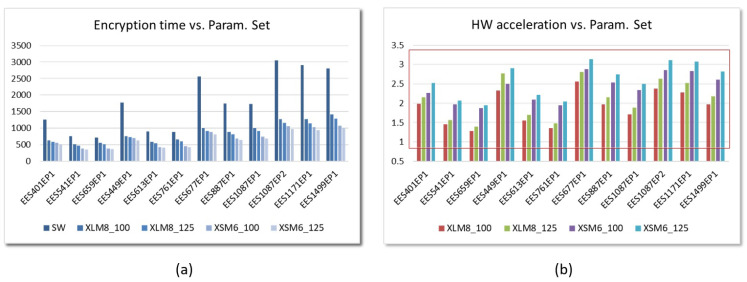
Times invested (**a**) and acceleration factors reached (**a**) in the encryption operation using the parameter sets defined in IEEE Std 1363.1 for SW and HW/SW embedded systems implemented on the Pynq-Z2 board (time displayed in μs).

This behavior is similar for embedded systems implemented on Zynq-7000 devices for all the parameter sets defined in the IEEE standard. Figure 21a shows the encryption times for SW and HW/SW implementations using AXI4-Lite and AXI4-Stream interfaces with M=8 and M=6, respectively, and operating frequencies of 100 and 200 MHz. As can be seen in Figure 21b, for the different sets of parameters, the acceleration factors range *between 1.7 and 2.8* for systems that use the MS2XL IP module and *between 2.0 and 3.1* for systems that use the MS2XS IP, operating in both cases at 125 MHz. Values within the rectangle in red indicate that HW acceleration is achieved.

The results are quite different when using the Ultra-96 board mainly due to two reasons. On the one hand, the ARM Cortex-A35 processors available in Zynq UltraScale+ devices operate 3.3 times faster than the ARM Cortex-A9 processors of Zynq-7000 devices, so only hybrid solutions using the fastest hardware accelerators could be competitive against pure software implementations. On the other hand, the aforementioned high read latency through the AXI4 bus in Zynq UltraScale+ devices greatly penalizes the use of hardware accelerators that incorporate the AXI4-Lite interface. As a consequence, regardless of the value of *M* and the operating frequency (which can reach 250 MHz in this device), no embedded system using the polynomial multiplier IP based on the AXI4-Lite interface is capable of providing an acceleration factor greater than 1 to implement the encryption operation with many of the standardized parameter sets.

**Figure 22:**
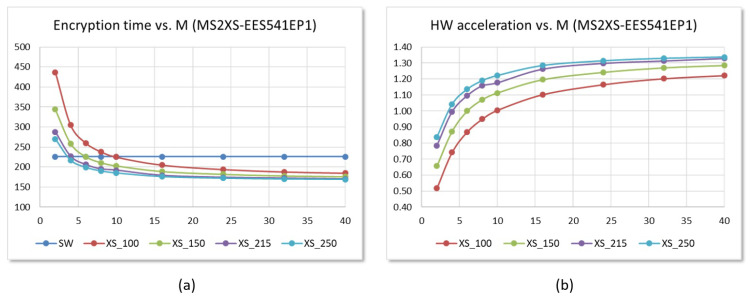
Evolution versus number of arithmetic units of the time invested (**a**) and the acceleration factor reached (**b**) in the encryption operation using the set of parameters EES541EP1 for SW and HW/SW embedded systems implemented on the Ultra-96 board using AXI4-Stream IPs (time displayed in μs).

The reduction in the times spent for loading coefficients and reading results in systems that use hardware polynomial multipliers with the AXI4-Stream interface does allow, in this case, for an improvement in the performance of the system over and above the increase in speed of the processing systems. Figure 22a shows the evolution versus *M* of the time invested in encryption operation for embedded systems that use the multiplier IP with the AXI4-Stream interface running at different operating frequencies and allows comparing its performance with that of a software implementation. As can be seen in Figure 22b, for the EES541EP1 parameter set, the use of hardware accelerators allows speeding up the encryption operation for values of M≥10 when a 100 MHz clock is used, M≥6 for 150 MHz, M≥2 for 215 MHz, and M≥1 if operating at 250 MHz. The acceleration factors compared to software implementation reach values *greater than 1.3* for this last operating frequency.

**Figure 23:**
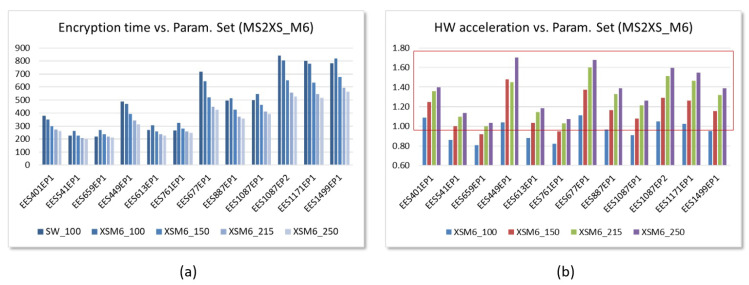
Evolution versus number of arithmetic units of the time invested (**a**) and the acceleration factor reached (**b**) in the encryption operation using the parameter sets defined in IEEE Std 1363.1 for SW and HW/SW embedded systems implemented on the Ultra-96 board using AXI4-Stream IPs (time displayed in μs).

The situation is similar for the other parameter sets defined in the IEEE standard, as illustrated in Figure 23a. The *enhancement factor* is *between 1 and 1.7* for systems with M=6 operating at 250 MHz. Finally, as Figure 23b shows, for the set of parameters in Table 1 that provide the same level of security, the lower the value of *N* and the higher the value of $d_{r}$, the greater the speed improvement introduced by the use of hardware accelerators.

## 8. Conclusions

This paper presents a hardware architecture to speed up polynomial multiplication that is used in the encryption and decryption operations of different variants of NTRU-based cryptographic schemes. The proposed solution contemplates the use of a variable number of arithmetic units to calculate several coefficients of the output polynomial in parallel, which allows designers to establish cost/performance compromises suitable for each application. This architecture is the basis of two IP modules, with standard interfaces AXI4-Lite and AXI4-Stream, respectively, that facilitate hybrid HW/SW implementations on Xilinx’s last generation programmable devices of the encryption and decryption operations defined in the NTRUEncrypt cryptographic scheme.

The work also provides a wide set of implementation results, which allows us to compare the different alternatives in terms of resources and execution times. This study considers the implementation of embedded systems in programmable devices of the Xilinx Zynq-7000 and Zynq-UltraScale+ families incorporating the two IPs and using the different parameter sets defined in IEEE Std 1363.1. The test programs were developed on the implementation platforms themselves using the facilities provided by the PYNQ environment. This strategy also made it easier to integrate hardware accelerators into the LibNTRU library to analyze the improvement achieved when the encryption operation is performed using hybrid solutions compared to pure software implementations running on the embedded system’s processor.

Finally, although the designs and results described in the paper are basically focused on the implementation of the currently standardized version of NTRUEncrypt, both the proposed architecture and methodology may also be very useful to facilitate future hardware implementation of other versions still under consideration in the context of the NIST PQC competition, in order to make them suitable for increasing the security of IoT devices.

## Figures and Tables

**Table 1:** Parameter sets for NTRUEncrypt.

Parameter Set	Recommended Security Level	*N*	*p*	*q*	$d_{f}$	$d_{g}$	dr
EES401EP1	112	401	3	2048	113	133	113
EES541EP1	112	541	3	2048	49	180	49
EES659EP1	112	659	3	2048	38	219	38
EES449EP1	128	449	3	2048	134	149	134
EES613EP1	128	613	3	2048	55	204	55
EES761EP1	128	761	3	2048	42	253	42
EES677EP1	192	677	3	2048	157	225	157
EES887EP1	192	887	3	2048	81	295	81
EES1087EP1	192	1087	3	2048	63	362	63
EES1087EP2	256	1087	3	2048	120	367	120
EES1171EP1	256	1171	3	2048	106	390	106
EES1499EP1	256	1499	3	2048	79	499	79

**Table 2:** Resources consumed by test systems using the two proposed multipliers with a multiplicity degree of 8 to implement the EES541EP1 parameter set on devices from the Xilinx Zynq-7000 and ZynqUltraScale+ families.

Zynq-7000	Slice LUTs (53,200)	Slice Registers (106,400)	Slice(13,300)	LUT as Logic (53,200)	LUT as Mem. (17,400)	Block RAM Tile (140)
MS2XL-M8 IP	743	195	267	743	0	8.5
MS2XS-M8 IP	603	90	209	603	0	8.5
MS2XL-M8 SoC	1094	652	409	1034	60	8.5
MS2XS-M8 So)	4346	5388	2811	3728	618	10.5
**Zynq UltraScale+**	**CLB LUTs** **(70,560)**	**CLB Registers** **(141,120)**	**CLB** **(8820)**	**LUT as Logic** **(70,560)**	**LUT as Mem.** **(28,800)**	**Block RAM Tile** **(216)**
MS2XL-M8 IP	637	194	145	637	0	8.5
MS2XS-M8 IP	517	90	116	517	0	8.5
MS2XL-M8 SoC	3094	2879	644	2891	203	8.5
MS2XS-M8 SoC	6343	8108	1262	5409	934	10.5

## Data Availability

Not applicable.
